# Feasibility Studies of Palm Oil Mill Waste Aggregates for the Construction Industry

**DOI:** 10.3390/ma8095319

**Published:** 2015-09-22

**Authors:** Jegathish Kanadasan, Auni Filzah Ahmad Fauzi, Hashim Abdul Razak, Paramananthan Selliah, Vijaya Subramaniam, Sumiani Yusoff

**Affiliations:** 1 StrucHMRS Group, Department of Civil Engineering, Faculty of Engineering, University of Malaya, Kuala Lumpur 50603, Malaysia; E-Mails: jegathish24@gmail.com (J.K.); auninostoc@gmail.com (A.F.A.F.); sumiani@um.edu.my (S.Y.); 2 Param Agricultural Soil Surveys Malaysia Sdn. Bhd., Petaling Jaya 46400, Malaysia; E-Mail: passparam@gmail.com; 3 Malaysian Palm Oil Board (MPOB), No 6, Persiaran Institusi, Bandar Baru Bangi, Kajang 43000, Selangor, Malaysia; E-Mail: vijaya@mpob.gov.my

**Keywords:** palm oil clinker, self-compacting mortar, feasibility, microstructure, sustainability

## Abstract

The agricultural industry in Malaysia has grown rapidly over the years. Palm oil clinker (POC) is a byproduct obtained from the palm oil industry. Its lightweight properties allows for its utilization as an aggregate, while in powder form as a filler material in concrete. POC specimens obtained throughout each state in Malaysia were investigated to evaluate the physical, chemical, and microstructure characteristics. Variations between each state were determined and their possible contributory factors were assessed. POC were incorporated as a replacement material for aggregates and their engineering characteristics were ascertained. Almost 7% of density was reduced with the introduction of POC as aggregates. A sustainability assessment was made through greenhouse gas emission (GHG) and cost factor analyses to determine the contribution of the addition of POC to the construction industry. Addition of POC helps to lower the GHG emission by 9.6% compared to control specimens. By channeling this waste into the construction industry, an efficient waste-management system can be promoted; thus, creating a cleaner environment. This study is also expected to offer some guides and directions for upcoming research works on the incorporation of POC.

## 1. Introduction

Although the agroindustry in Malaysia has been the backbone of the country for a few decades, products from this industry generate a huge amount of waste that requires suitable disposal. Palm oil factories generate various types of waste, which include, oil palm fiber, oil palm shell (OPS), palm oil mill effluent (POME) and empty fruit bunches (EFB). Improper management of these wastes could lead to environmental pollution. Hence, introducing the 3R (Recycle, Reuse, Reduce) concept could help to save the environment from pollution besides supporting the sustainability of certain industries. Incorporation of these waste products would also help to sustain those natural resources that are rapidly depleting and support the global push towards “green” production. Al-Oqla and Sapuan [1] reported that date palm fiber has the ability to produce higher specific modulus of elasticity to cost ratio, which are suitable for use in automotive industry. Utilization of dry composite electroplating sludge (CEPS) in the form of green pigment for the production of cement based decorative mortar showed almost identical compressive strength, tensile bond strength and flexural strength compared to reference concrete besides having lower heavy metal mortar leachates [2]. Use of sugar filter mud (FM) lower than 20% as lime based raw material in cement depicted higher compressive strength behavior compared to reference concrete while further increase leads to a decrease in strength [3]. Incorporation of 5% kenaf core in polyurethane composites leads to more porous and lower density composites. However, this material improves the thermal resistance properties of the composites [4]. The utilization of waste byproducts can help to improve the waste management to reduce environmental pollution. Channeling these waste products to other industries to be used would be a creative and resourceful method to increase the productivity of the respective industries. Substitution of cement and fine aggregates with unprocessed lignite-coal fly ash and rice husk ash (RHA), respectively, could help to bring down the cost of the self-compacting concrete (SCC) [5]. Akram *et al.* [6] found that addition of baggase ash reduced the cost of SCC by 35.63% when compared to reference concrete. From an economic viewpoint, the agricultural industry can save cost through the appropriate disposal of these abundantly available byproducts. The burning process of OPS and oil palm fiber produces palm oil clinker (POC), which is obtained in large chunks ranging between 100 and 400 mm before being crushed into aggregates with the required sizes. Physically the inner portions of POC are highly porous, which significantly contributes towards the lightweight nature.

Utilization of POC in the construction industry could be a step in the right direction to substitute the depleting natural aggregates as well as providing proper waste management in the agricultural industry. In 2013, 5.23 million hectares of oil palm were planted, which was 3% higher than in 2012 [7]. In July 2014, there were 440 fresh fruit bunch (FFB) mills in Malaysia, of which 247 mills were located in Peninsular Malaysia and the rest in Sabah and Sarawak [8]. From the different geographical conditions and locations in each state, there could be some variation in terms of the physical and chemical properties of the POC obtained, which needs to be determined. The variation could be due to the burning temperature, incineration ratio of shell and fiber or the type of soil. Notwithstanding, the variations in the daily operation between the mills, these feed materials have considerable calorific and economic value and have high potential to be utilized in other industries. Considering the numerous amount of contributing factors to varying POC characteristics it is vital that some of the key fresh and hardened properties undergo test evaluation.

The plantation crops in Malaysia have grown to become one of the country’s main contributors to the economy. Over the years, the agricultural industry has expanded with thousands of hectares of land being planted with oil palm, rubber, paddy, cocoa, coconut and others. Studies show that almost 57 million tons of palm oil was produced in total by Malaysia and Indonesia in 2012, which is 85% of the global production [9,10]. However, each phase of palm oil production generates an immense amount of waste, which needs proper disposal. POC is obtained as a waste byproduct at the final stages of burning in boilers. Usually mesocarp fiber and shell are burned in the boilers to produce steam to generate electricity [11]. Currently POC is either landfilled or utilized as cover for the roads leading to the oil palm estates. A number of studies have been conducted on the incorporation of waste agricultural byproducts in the construction industry. Researchers reported that using granulated corn cob produced satisfactory material properties when mixed at 6:1:1 (corn cob granulate:Portland cement:water) ratio and may be suitable for non-structural applications [12]. Sales and Lima [13] found from their studies that using sugarcane bagasse ash as a replacement for sand at most of the levels exhibited strength values greater than the control specimens.

When Portland cement was substituted with 20% black rice husk ash (BRHA), the leaching of concrete reduced for HCl and H_2_SO_4_ attack [14]. The utilization of 20% banana leaf ash (BLA) to replace cement with a water binder ratio of 0.5 was found to produce concrete with a strength of 48 ± 2 MPa at 28 days, which was 12% greater than the control specimens [15]. When bagasse ash was used to replace cement, it was found to reduce the chloride penetration rate [16]. Besides agricultural waste, industrial waste is also being converted into useful sources of aggregates. Senthamarai *et al.* [17] reported that the addition of ceramic electrical insulator waste as a substitute for coarse aggregate produced similar permeation characteristics to that of normal concrete. Researchers report that the utilization of superfine powder from granitic quarry sludge waste in mortar enhances the density of the matrix to elevate the performance of chloride resistance by 70% compared to the control mix [18]. In their research, Tan and Du [19] found that the incorporation of waste glass as a substitute for fine aggregate produced lower drying shrinkage of the mortar to enhance the dimensional stability. Use of wollastonite as cement replacement material between 10% and 15% enhanced the strength and durability properties of concrete [20].

The focus on lightweight concrete has grown substantially over the years due to its many advantages. POC aggregate used for concrete production is also known to produce lightweight concrete with a density below 2000 kg/m^3^ [21]. Wang [22] reported that at 91 days, each self-compacting lightweight aggregate concrete produced chloride penetration below 1000 Coulombs, thereby indicating good resistance against chloride intrusion. Chen *et al.* [23] observed that 70% substitution of recycled green building materials compared to that of the control ones resulted in a 1.8 times increase in the resistance of the concrete, which may be due to the insulating properties of recycled green building materials. The incorporation of sewage sludge as a lightweight aggregate has the ability to produce lightweight concrete with a density of 1400–1500 kg/m^3^, compressive strength above 15 MPa and flexural strength above 3 MPa [24]. Using water treatment sludge as lightweight aggregate has the ability to produce concrete with satisfactory compressive strength and splitting tensile strength properties [25]. The utilization of municipal solid waste incineration fly ash and reaction ash with reservoir sediment produced lightweight concrete with a strength of 40 MPa with 63 MPa mortar strength despite having only 6 MPa aggregate crushing strength [26]. Researchers have reported that the thermal conductivity and diffusivity are reduced when proportion of lightweight aggregate incorporated increased [27]. Researchers have found that when fly ash cenospheres are replaced completely with iron ore tailings, the thermal conductivity of the green lightweight engineered cementitious composites produced was 21% lower on average [28]. Self-compacting lightweight concrete mixes with 10% and 15% of metakaolin attained a “very low” category of chloride ions ingress compared to “low” category for 0% and 5% [29]. A greater compressive strength and ultrasonic pulse velocity (UPV) value was observed when 20% of cathode ray tube (CRT) glass was utilized with 5% limestone powder (LS) [30]. According to Bogas *et al.* [31], addition of recycled lightweight concrete aggregates produced greater structural efficiency values, leading towards high sustainability concrete. From the environmental point of view, previous study carried out by Kanadasan and Abdul Razak [32] showed that utilization of POC in concrete reduces the carbon emission by almost 23% compared to normal concrete. Table 1 tabulates the review on important findings of using waste materials in the construction industry.

**Table 1:** Review on important findings of using waste materials in the construction industry.

Reference	Type of Waste	Content	Findings
Pinto, Vieira, Pereira, Jacinto, Vilela, Paiva, Pereira, Cunha and Varum [12]	Agricultural waste	Granulated corn cob	Suitable for non-structural purposes
Sales and Lima [13]	Agricultural waste	Sugarcane bagasse ash	Strength values greater than the control specimens
Chatveera and Lertwattanaruk [14]	Agricultural waste	Black rice husk ash (BRHA)	Leaching of concrete reduced for HCl and H_2_SO_4_ attack
Kanning, Portella, Bragança, Bonato and dos Santos [15]	Agricultural waste	Banana leaf ash	Concrete with a strength of 48 ± 2 MPa at 28 days
Kanadasan and Abdul Razak [32]	Agricultural waste	Palm oil clinker (POC)	Reduces the carbon emission by almost 23%
Senthamarai,Manoharan and Gobinath [17]	Industrial waste	Ceramic electrical insulator waste	Similar permeation characteristics to that of normal concrete
Ramos, Matos, Schmidt, Rio and Sousa-Coutinho [18]	Industrial waste	Superfine powder from granitic quarry sludge waste	Elevate the performance of chloride resistance by 70% compared to the control mix
Kalla, Rana, Chad, Misra and Csetenyi [20]	Industrial waste	Wollastonite	Enhanced the strength and durability properties of concrete
Chen, Wang and Tang [26]	Industrial waste	Municipal solid waste incineration fly ash and reaction ash with reservoir sediment	Lightweight concrete with a strength of 40 MPa with 63 MPa mortar strength

In this study, POC samples from all 12 states throughout Malaysia were investigated for their physical and chemical properties besides carrying out some engineering property tests to study the feasibility of utilizing POC in mortar. A palm oil mill was randomly selected to represent the respective state. Obtained POC was replaced as fine aggregates in mortar. A fresh property test was carried out to ensure they are within the high flowability mortar region to satisfy the properties for self-compacting mortar (SCM). Mechanical property tests were undertaken to investigate the compressive and flexural strength. Microstructure studies were also carried out on mortar specimens to further investigate the characteristics and interface of POC with the cement paste. Sustainability aspects were evaluated through energy requirement, cost factor and greenhouse gas emissions. This study will provide some insight and guide to upcoming research works to further enhance the understanding of the physical and chemical characteristics of POC.

## 2. Experimental Procedure

### Materials, Mix Proportion and Test Methods

Ordinary Portland cement (CEM I) 52.5N was utilized in this investigation. The chemical composition of cement is shown in Table 2. A poly-carboxylate based superplasticiser (SP) was used with a density of 1.08 g/L as given by the manufacturer. Table 3 tabulates the mix proportion for mortar specimens with the POC collected from all states in Malaysia. The complete chemical and physical characteristics test were carried out on the POC aggregate obtained from all the states. Dry mixing is carried out for 2 min before mixing with water and SP for another 3 min. The mixing time is maintained to be within 5 min throughout the study. Tests on the fresh properties were carried out to evaluate the self-compacting behavior of mortar mixes through the slump flow and mini V-funnel tests. In addition, the hardened properties were evaluated by means of density, compressive and flexural strength tests. Mortar cubes of 50 mm size and 40 mm × 40 mm × 160 mm flexural beams were prepared and water cured for 28 days under normal room temperature in this study. Mortar fragments were also examined under a scanning electron microscope to study the morphology in the hardened state.

**Table 2:** Chemical composition of ordinary Portland cement (CEM I) 52.5N.

Oxides	CEM I 52.5N
Al_2_O_3_	5.37
CaO	64.00
Fe_2_O_3_	2.94
K_2_O	0.17
MgO	3.13
Mn_2_O_3_	0.24
Na_2_O	0.12
SiO_2_	20.29
SO_3_	2.61
P_2_O_5_	0.07
TiO_2_	0.12
Others	0.94
Loss on ignition	1.40
**Bogue Compound Composition of Cement (Compound)**	**% by Mass**
C_2_S	13.95
C_3_A	9.26
C_3_S	58.62
C_4_AF	8.95

**Table 3:** Mix proportion.

State	Abbreviation	Cement (kg)	Water (kg)	POC Fine (kg)	Water/Cement Ratio	Superplastisizer Dosage (%)
Kedah	KDH	2.28	0.64	2.509	0.28	0.4–0.6
Kelantan	KLT	2.28	0.64	2.036		
Terengganu	TRG	2.28	0.64	2.329		
Penang	PNG	2.28	0.64	2.396		
Perak	PRK	2.28	0.64	2.374		
Pahang	PHG	2.28	0.64	2.576		
Negeri Sembilan	NSE	2.28	0.64	2.621		
Selangor	SEL	2.28	0.64	2.374		
Melaka	MLK	2.28	0.64	2.565		
Johor	JHR	2.28	0.64	2.486		
Sabah	SBH	2.28	0.64	2.340		
Sarawak	SWK	2.28	0.64	2.430		
Selangor	NML *	2.28	0.64	2.925 (Sand)		

* NML—River sand obtained from a source in state of Selangor.

## 3. Results and Discussion

### 3.1. Physical and Chemical Properties of POC

**Figure 1:**
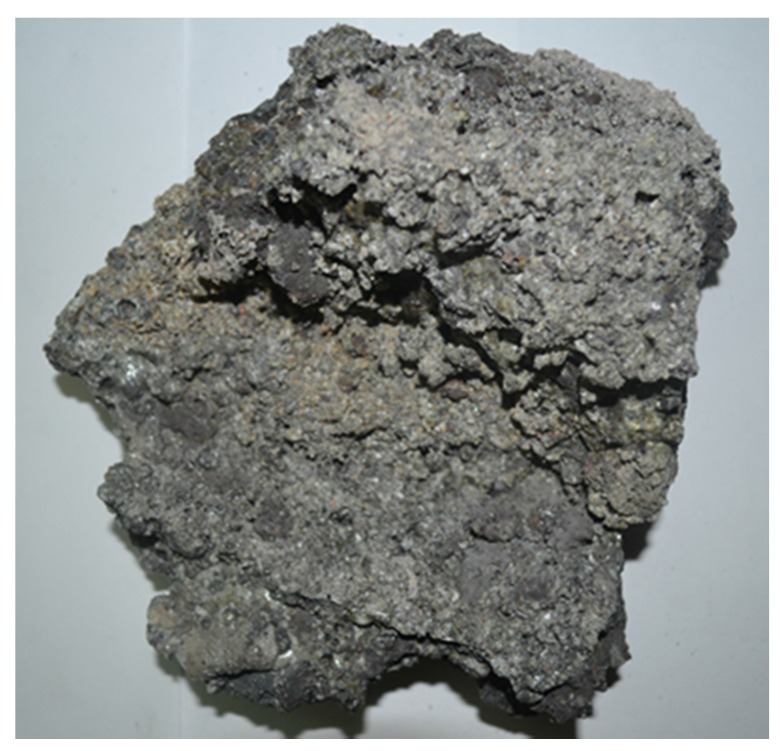
A large chunk of palm oil clinker (POC).

**Figure 2:**
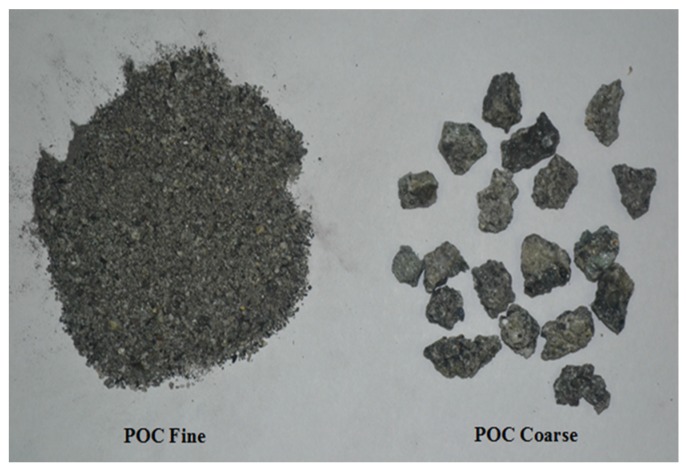
POC Fine and POC Coarse.

**Figure 3:**
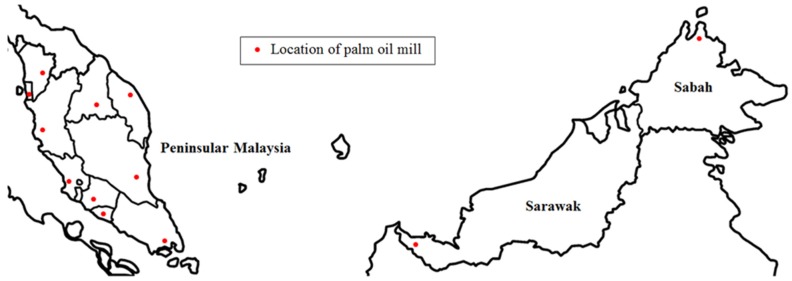
Location of palm oil mills selected for POC sampling in Malaysia (Map from www.Mapsof.net).

**Figure 4:**
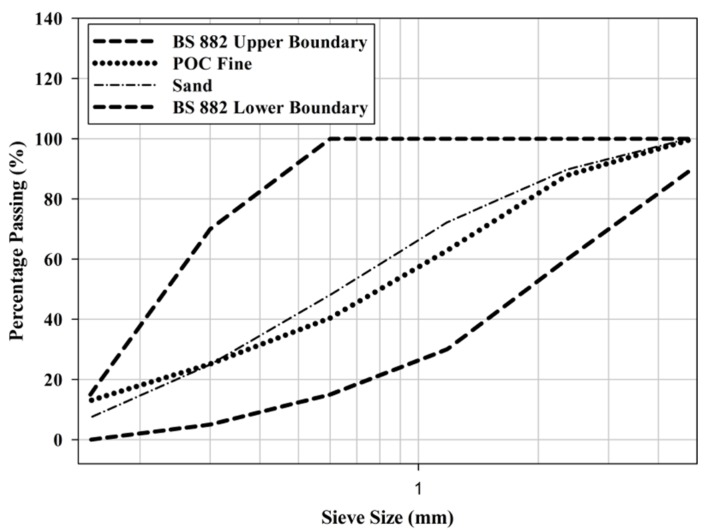
Particle size distribution for sand and POC fine.

**Figure 5:**
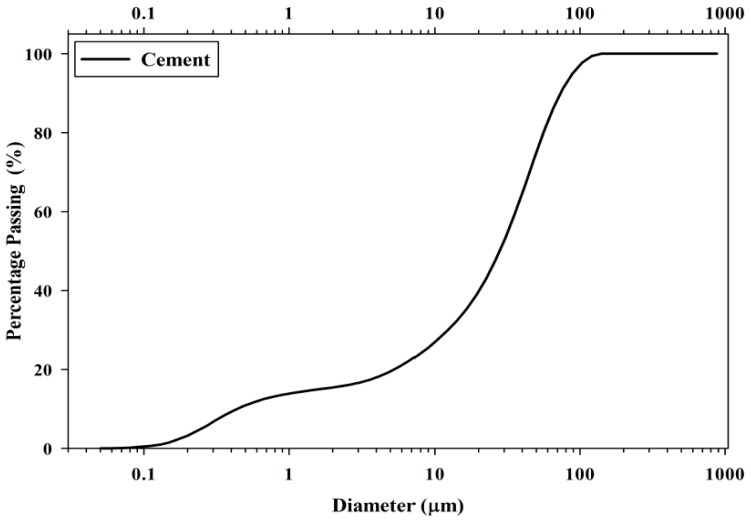
Particle size distribution for ordinary Portland cement (CEM I) 52.5N.

Figure 1 shows a large chunk of POC collected from the mill, while Figure 2 exhibits the POC fine and POC coarse, which were prepared for this study. A palm oil mill was randomly chosen in each state in Malaysia to represent the POC samples of that particular state. The POC samples were collected from 12 locations in Malaysia including Sabah and Sarawak. Figure 3 shows the locations of the palm oil mill selected from each state for this study. Figure 4 shows the difference in the particle size distribution between sand and POC fine. The similar grading features of both the curves indicate the suitability of POC fine substitution. Figure 5 depicts distribution curve for CEM I. The physical properties of the POC materials collected are listed in Table 4. They are determined according to BS 812-2:1995 [33] and BS 812-109 [34]. X-ray fluorescence (XRF) test was carried out on the sample of each state to identify the variation in the sample’s chemical content. The chemical compositions of the POC samples from the different states are depicted in Table 5. These values are based on an average XRF test result for every sample collected. There are some evidences of variation among the POC samples, most notably in terms of silica (SiO_2_) content. This oxide component could have a significant effect on the engineering properties of SCM produced. Despite the large difference among the samples from each state, generally, the POC contains between 60% and 75% silica (SiO_2_).

**Table 4:** Physical characteristics of the aggregate.

State	Abbreviation	Specific Gravity	Water Absorption (%)	Moisture Content (%)
Kedah	KDH	2.23	1.93	0.17
Kelantan	KLT	1.81	4.12	0.25
Terengganu	TRG	2.07	2.28	0.04
Penang	PNG	2.13	3.05	0.07
Perak	PRK	2.11	1.40	0.04
Pahang	PHG	2.29	2.34	0.03
Negeri Sembilan	NSE	2.33	4.05	0.31
Selangor	SEL	2.11	4.10	0.48
Melaka	MLK	2.28	1.65	0.02
Johor	JHR	2.21	2.37	0.17
Sabah	SBH	2.08	2.54	0.05
Sarawak	SWK	2.16	5.67	0.04
Selangor	NML *	2.60	1.59	0.07

* NML—River sand obtained from a source in state of Selangor.

**Table 5:** Chemical composition of the POC samples from different states.

State	SiO_2_	K_2_O	CaO	SO_3_	Fe_2_O_3_	Al_2_O_3_	MgO	P_2_O_5_	TiO_2_	Na_2_O	Loss on Ignition
KDH	65.10	9.23	3.89	0.16	6.34	3.28	2.34	3.06	0.13	0.07	0.91
KLT	73.31	8.78	4.01	0.13	6.13	6.00	2.83	1.43	0.28	0.09	−0.59
TRG	72.64	9.65	4.42	0.17	3.89	5.18	2.56	3.96	0.17	0.01	0.16
PNG	69.91	9.24	8.56	0.14	5.15	4.15	3.92	3.24	0.15	0.12	−0.12
PRK	74.29	6.22	5.10	0.15	2.09	3.11	1.72	2.79	0.15	<0.1	0.49
PHG	60.79	5.17	10.88	0.18	15.64	7.27	2.23	1.61	0.27	0.13	−0.89
NSE	65.64	7.26	4.11	0.25	14.41	7.56	2.64	1.73	0.29	0.08	−0.26
SEL	64.84	12.82	5.96	0.16	4.19	3.42	5.01	3.37	0.12	0.09	1.54
MLK	57.41	11.32	6.95	0.22	10.11	4.95	4.01	4.90	0.17	0.12	<0.1
JHR	69.05	11.09	5.70	0.19	3.71	4.73	2.27	3.22	0.23	<0.1	0.78
SBH	62.45	13.48	9.51	0.21	2.20	1.42	6.09	7.33	0.07	0.05	0.35
SWK	62.52	8.44	16.74	0.23	1.10	0.82	3.43	4.75	0.08	0.11	0.28
Mean	66.50	9.39	7.15	0.18	6.25	4.32	3.25	3.45	0.18	0.09	0.23
Standard Deviation	5.13	2.40	3.63	0.04	4.54	1.99	1.24	1.59	0.07	0.03	0.64

There are few possible reasons behind these variations. Although most of them are qualitative, they have to be taken into consideration to understand the major contribution to the changes. These qualitative assessments are to highlight and characterize the POC as it is vital to understand the source of variation. This will assist in optimizing the waste material for usage in various industries. One of the variations could be due to the burning temperature of POC in the boiler. Most of the mills set their burning temperature between 600 and 900 °C, which could result in the variation in the POC properties during the pyrolysis process. At a lower temperature, there is a high possibility that the pyrolysis process is incomplete and that some shells and fiber may remain as unburned residue.

Secondly, the addition of shell and fibers of different proportions produces POC with a different chemical composition. The proportion of shell and fiber fed into the boiler depends on the mill’s practice and policy with regards to environmental concern. Vijaya, Ma, Choo and Nik Meriam [11] also reported that some of the mills vary their feed ratio of shell and fiber taking into consideration of the economic value to sell them off as fuel for the use in other industries.

According to available sources, there are more than 50 types of soil series in Malaysia and this also has a contributory effect on the composition of the POC obtained. There is a vast range of soil types existing in the area where the oil palm plantations are geographically located depending on their proximity to the coastal or highland areas. The types of soil within a radius of approximately 100 km from the mill are assumed to have an influence on the quality of the FFB obtained from different plantation areas. Table 6 tabulates all the types of soil and their classification obtained through Paramananthan [35]. Although there is a variation in the dominant type of soil present in each state, generally they are fine clayey, fine loamy or fine silty. Further detailed studies are required to establish the relationship between the types of soil and the quality of the crop, which could help to optimize the quality of FFB obtained as well as help to quantify the properties of the waste materials produced.

**Table 6:** Types of soil at various sampling points.

State	Soil Classification
KDH	Clayey-Skeletal (>35% gravel within 50 cm depth, >35% Clay)
KLT	Fine silty (35%–60% Clay, >30% silt)
TRG	Fine clayey (35%–60% Clay, <30% silt). Moderately deep (50–100 cm) soil
PNG	Clayey (>35% Clay)
PRK	Very fine (>60% Clay)
PHG	Fine clayey (35%–60% Clay; <30% silt)
NSE	Fine silty (35%–60% Clay, >30% silt)
SEL	Fine clayey (35%–60% Clay, <30% silt)
MLK	Fine clayey (35%–60% Clay; <30% silt)
JHR	Fine loamy (18%–35% Clay)
SBH	Fine sandy clay (35%–60% Clay; <30% silt)
SWK	Fine loamy (18%–35% Clay)

**Figure 6:**
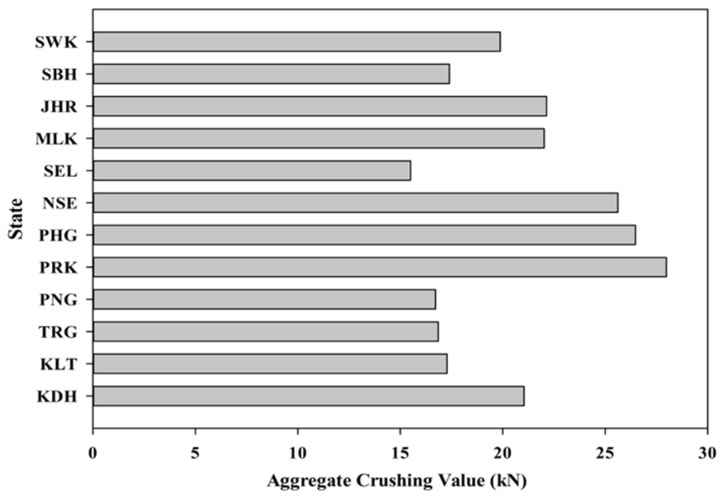
Aggregate crushing values for various states.

The aggregate crushing value (ACV) test is another important test that was carried out to physically characterize the coarse aggregate. Figure 6 depicts the variation in the crushing values of the POC aggregates from various states in Malaysia. As POC aggregate is very much porous and lighter in nature, the load bearing capacity may be significantly affected. The highly porous nature of the POC aggregate will induce crack propagation much easier than dense cement paste. This will result in the aggregate failing much earlier than the hardened cement paste. Thus, the ACV value significantly relates the strength carrying capacity of the mortar samples under load. The test was conducted based on BS 812-110 [36] and BS 812-111 [37]. Generally, the ten percent fines (TFV) of the ACV values for POC are between 15 and 30 kN. A higher crushing value generally indicates the high capability of the aggregate to sustain load, which indirectly provides good strength achievement in the mortar. Comparing the trend of ACV for the samples of each state, generally, the states with higher ACV test results gave higher compressive strength results. This relationship could also serve as a potential indicator or estimator of the strength properties for different POC samples.

### 3.2. Mineralogical Properties

**Figure 7:**
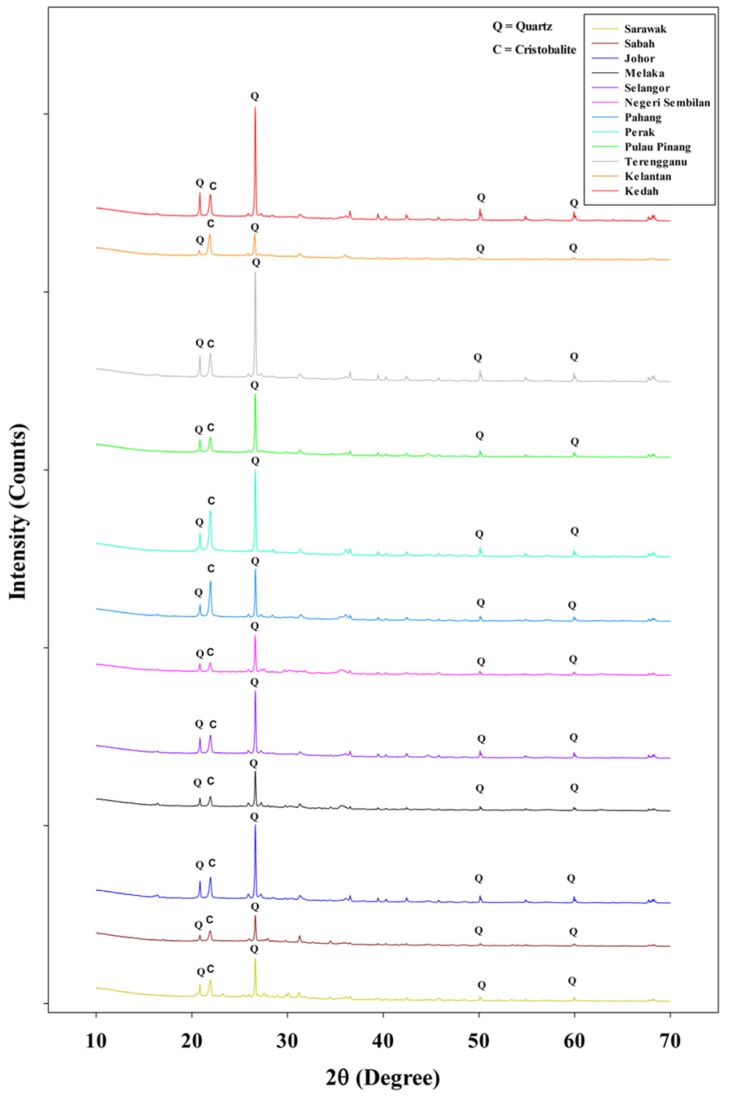
X-Ray diffraction results for all states.

Mineralogical analysis on the POC samples from all the states in Malaysia was conducted using X-ray diffraction (XRD) and the results are presented in Figure 7. The XRD plots illustrate that the POC samples consist predominantly of quartz as apparent by the presence of peaks at 2θ of 20.83°, 26.61°, 50.11° and 59.93°. In addition, crystallized phase of cristobalite was also detected through the presence of a peak at 2θ of 21.92°. Despite having varying levels of intensity, the XRD patterns for the different states were consistent indicating the type of minerals present in all states are similar.

### 3.3. Microstructure of POC Aggregate

**Figure 8:**
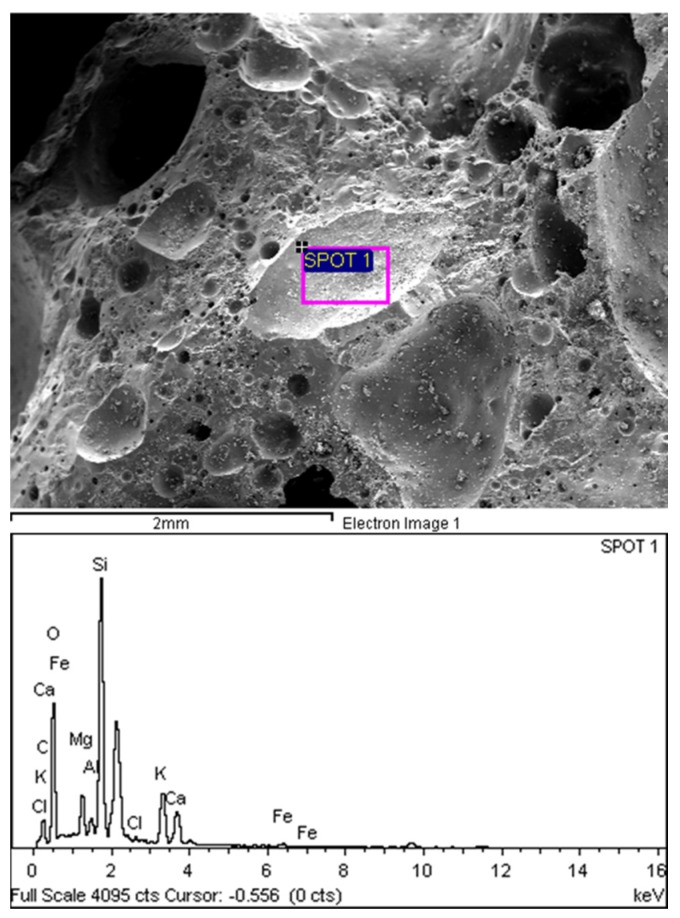
POC aggregate micrograph at a larger magnification.

**Figure 9:**
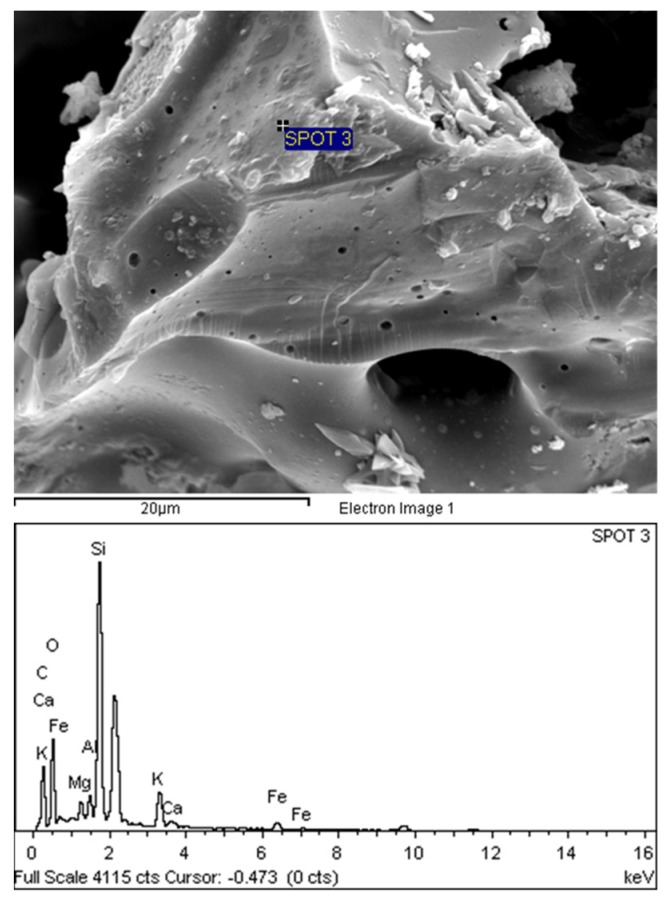
Presence of micro-pores within internal POC aggregate structure.

Figure 8 shows a scanning electron micrograph (SEM) with energy dispersive X-ray spectroscopy (EDX) for POC specimens. As observed, the porous nature of POC appears to be eminent at a larger magnification. Irregularities in the shape of the POC can also be seen. A large number of voids exist on the surface ranging on average from 1 mm to 500 μm. Figure 9 shows the micro-pores that exist on the surface of the POC aggregate at 20 μm magnification. The existence of the large number of voids and pores contributes significantly to the light nature of POC aggregate. These pores have a significant effect on the load carrying capacity of the mortar. In addition, the inconsistent shape of the POC aggregate may also affect the aggregate-to-aggregate packing level and aggregate-paste boundary. From EDX study, the graphs obtained confirmed the chemical traces which were reported earlier through chemical composition whereby significant traces were detected for silica, aluminum and magnesium and iron.

### 3.4. Fresh Mortar Properties

**Figure 10:**
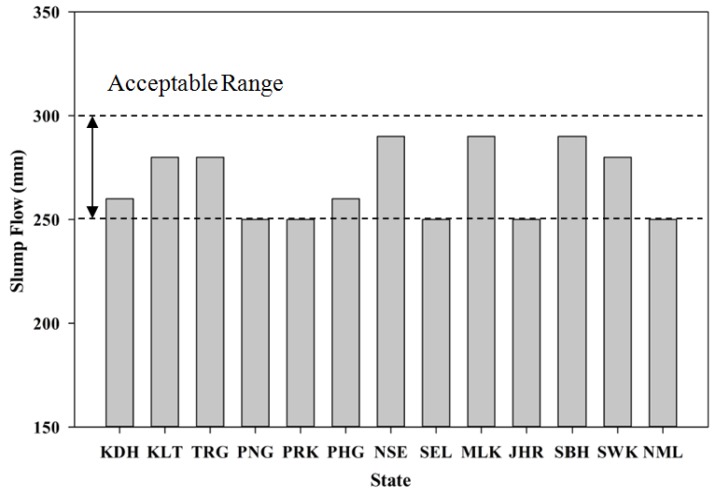
Slump flow test results.

SCM, which involves a balance between flowability, passing ability and segregation resistance, has to be checked with the stipulated standard to ensure that it meets the requirements. The tests carried out include the slump flow and V-funnel which was outlined by European Federation of National Associations Representing producers and applicators of specialist building products for Concrete (EFNARC) [38]. All the test limits have to be satisfied to ensure maximum performance of SCM. SCM is preferred as it provides a better coating around the POC aggregate, which could improve the performance of the mortar specimens. In addition, SCM also tends to improve the packing level of the fresh mortar to improve the bond between the irregular and porous characteristics of POC with the cement paste. Figure 10 shows the variation in slump flow for the samples of each state. The slump flow values were kept between 250 and 300 mm. This range was selected to achieve a flowable mortar to satisfy the self-compactability and high flowability requirements. The lightweight nature of POC aggregate also plays a vital role in exhibiting a larger slump flow. The much lighter POC reduces the mass of the overall mortar skeleton to allow for the maximum flowability. Previous study conducted on two types of lightweight aggregate confirms that the lighter ones produced a higher slump range [39].

**Figure 11:**
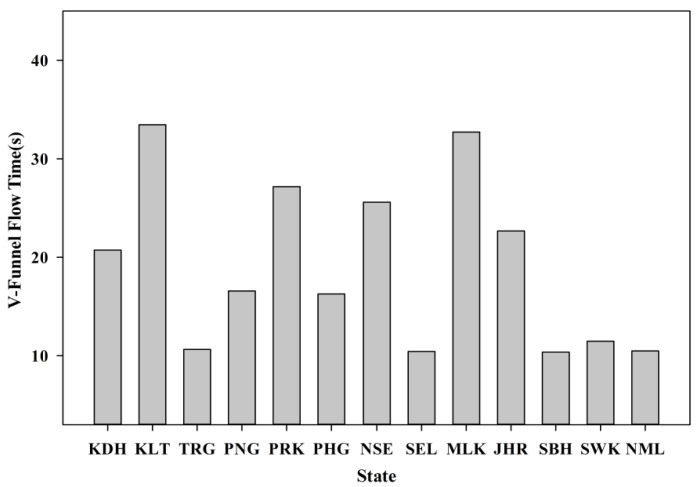
V-Funnel flow time results.

Figure 11 depicts the relationship between the V-funnel flow time and the POC samples from the various states. Generally, to satisfy the requirement for a high flowable mix, the flow time was kept between 10 and 35 s. However, the physical characteristics of the POC aggregate play a vital role in producing different mix viscosities, thus giving a range of flowability. The sharp and rough surface may affect the self-compacting properties for different state POC mixtures.

### 3.5. Engineering Properties

#### 3.5.1. Density

**Figure 12:**
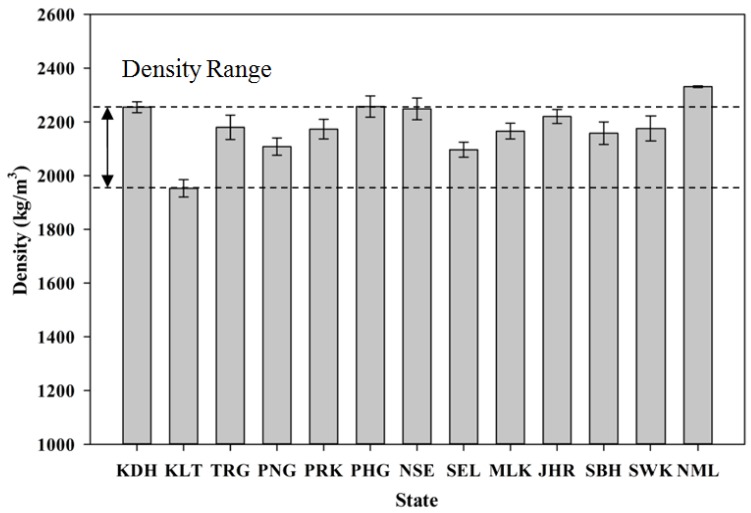
Variation in density for the samples of each state.

Figure 12 shows the variation in density for the samples for each state. Generally, they lie between 1950 and 2260 kg/m^3^, which can be classified as lightweight. In general, an approximate reduction of about 7.0% can be observed when the mortar specimens are fully replaced with POC. Compared to normal weight mortar, POC incorporated mortar specimens are much lighter. The lower specific gravity created by the high porosity and void content of POC aggregate contributes significantly to the lower density of the overall mortar specimen. The introduction of POC also concurrently reduces the load bearing capacity of the mortar, which can be evaluated through its compressive strength.

#### 3.5.2. Compressive Strength and Structural Efficiency

**Figure 13:**
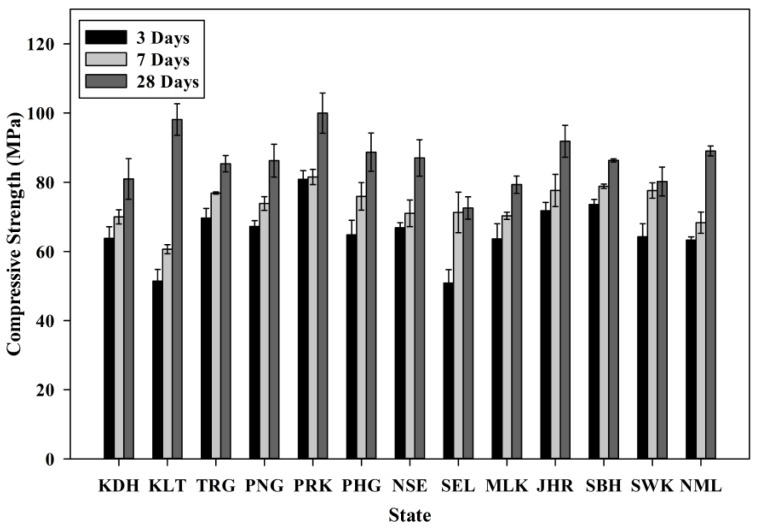
Compressive strength for all states.

**Figure 14:**
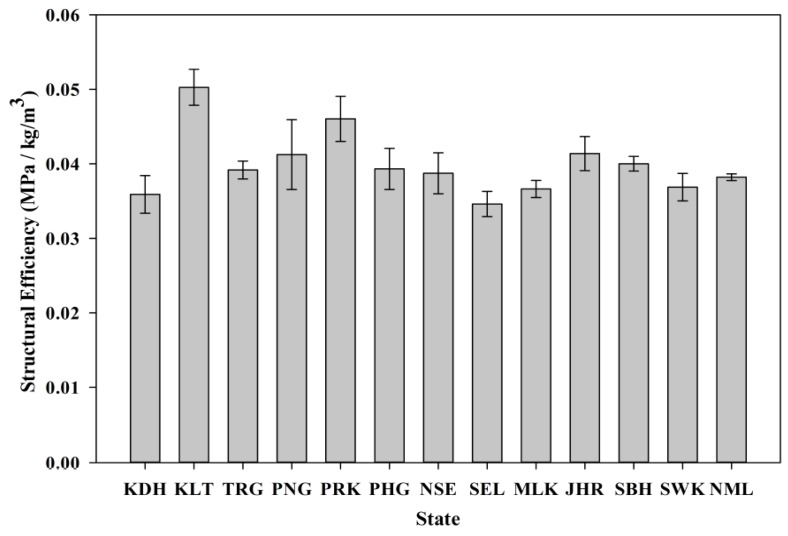
Structural efficiency for all states.

The compressive strength of SCM incorporating POC is presented in Figure 13. The test was carried out based on BS EN 12390-3 [40]. A reduction of strength was observed when the POC was incorporated in the mixes. The ACV value greatly affects the load bearing capacity of the POC aggregate. In addition, the highly porous nature with micro voids within the internal structure decreases the density of the aggregate. The availability of empty space allows for quick crack propagation as well as failing rapidly under applied load. In addition, the structural efficiency factor plays an important role in relating the strength and density to obtain a good and reliable indicator of the effectiveness of lightweight structures. Figure 14 shows the structural efficiency for the samples for each state. This concept was introduced to provide a similar platform for comparing the lightweight and normal weight mortar specimens [41,42,43]. This evaluation was performed by creating a ratio between the density and compressive strength of the respective mixes. The lower density of POC mixes reduces the capability of the hardened specimens to sustain high load. As in this research, the higher strength properties of POC aggregate mortar compared to its density produced greater structural efficiency values. All the samples were within a range between 0.035 and 0.05 MPa/(kg·m^−3^). The strength properties obtained are also closely related to the physical characteristics of the samples for each state.

Despite having these major variations not only in the physical but also chemical properties, the strength of most POC samples surpassed 70 MPa at 28 days indicating a satisfactory strength sustaining capability. The presence of SiO_2_ may also provide some indication concerning the possible strength achievement for mixes incorporating POC. The Perak and Kelantan specimens, which recorded the highest compressive strength and structural efficiency, actually possessed higher SiO_2_ values than the samples of the other states. The variation between the SiO_2_ values is closely related to the three major factors discussed earlier.

#### 3.5.3. Flexural Strength

**Figure 15:**
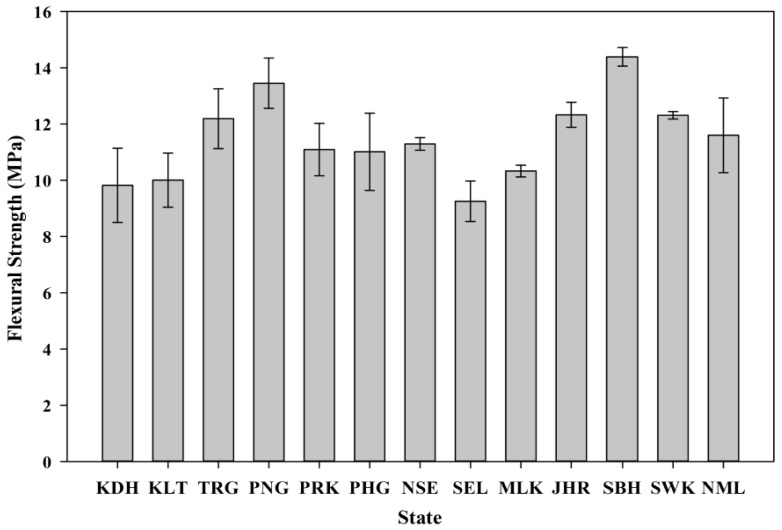
Flexural strength results for all states.

The mortar samples of each state were subjected to flexural load to evaluate the performance of the POC. The test was performed according to ASTM C348 [44]. The distance between the supports is 100 mm. Figure 15 shows the flexural strength results at 28 days for each state. Although there was a significant variation in terms of the physical and chemical properties of the POC samples, the flexural strength results were not significantly affected. Most of the strength achievements were beyond 8 MPa at 28 days. The lower flexural values for samples incorporating POC may be attributed to the higher ACV values, which induce premature failure when subjected to flexural load compared to other samples. The internal pores within the POC aggregate may induce early crack propagation across mortar specimens to allow the aggregate to fail faster compared to the cement paste. In addition, the irregular shape of the POC also could affect the interface between the paste and the aggregate.

#### 3.5.4. Microstructure (POC Aggregate in Mortar)

**Figure 16:**
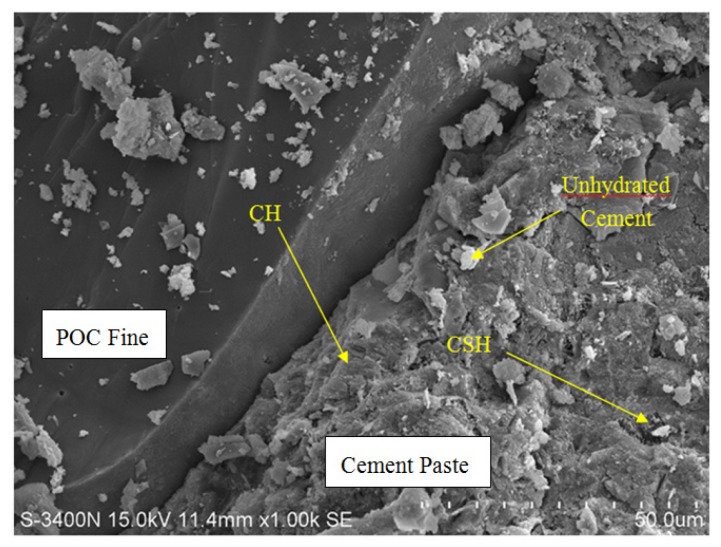
Aggregate cement paste interface.

A sample was taken to investigate the interface between the POC aggregate and the cement matrix through scanning electron microscopy (SEM). The sample was randomly selected to observe the formation of the hydrated products and bond between the aggregate and the paste. It was observed from the SEM examination that there is normal formation of the calcium hydroxide and calcium silicate crystalline phases as evident in other ordinary Portland cement mortar. Figure 16 shows the aggregate and cement paste interface. Under low magnification, the bond between the aggregate and the cement paste is quite evident. Detachment of the POC aggregate from the cement paste is also apparent. Micro-pores can also be seen on the POC aggregate. The spectrums produced through EDX are shown below each SEM image.

**Figure 17:**
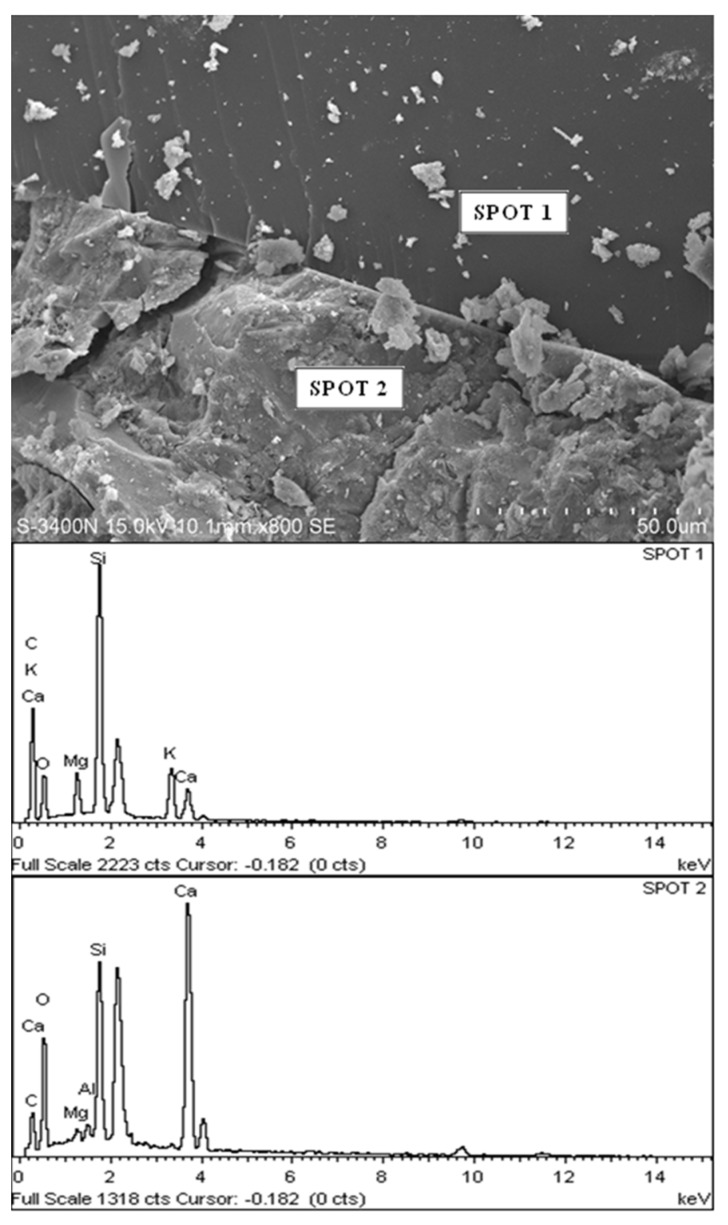
POC aggregate—Cement paste interface with X-ray spectroscopy (EDX).

Figure 17 shows the SEM image and EDX results for the specimens from Sabah. From the EDX results, the higher silica (Si) (SPOT 1) and calcium (Ca) (SPOT 2) peaks corresponding to the POC aggregate and cement paste, respectively, indicate the boundary between the aggregate and the cement paste. The irregular nature of the POC aggregate, which is porous and has sharp edges, creates a rough surface texture. Previous researchers report that when the roughness of the aggregate is high, the bond strength of the interface may increase [45]. As the mortar specimen produced is based on SCM, the high paste volume significantly affects the interlocking configuration between the POC and the paste. From the SEM images, it is also clear that the packing level of the aggregate and paste are quite high which relates to the properties of the SCM, promoting a packed mortar structure. Previous studies reported that SCC generally reduces the void ratio on interfacial transition zone (ITZ) and enhances the homogeneity for uniform distribution of pores [46].

### 3.6. Sustainability Components

The major highlight of this study is the introduction of POC, a waste material originating from oil palm mills, for concreting work. Despite having an abundant supply from palm oil mills, POC is mostly utilized as cover for potholes on the roads leading to the palm oil estates. As the nature of POC is lightweight and porous, it could serve well as a lightweight aggregate for mass mortar and concrete production. Kanadasan and Razak [21] reported that based on the ACV values, POC aggregate required three times less energy to produce relative to ordinary gravel. Looking from the energy efficiency point of view, POC will help to reduce the need for excessive utilization of energy in the preparation stages. This may be beneficial to certain applications for which the energy and cost savings are vital. Incorporation of POC will not only lower the energy requirement but also significantly reduces the electricity consumption and cut down the processing time compared to the required materials from the natural resources.

**Figure 18:**
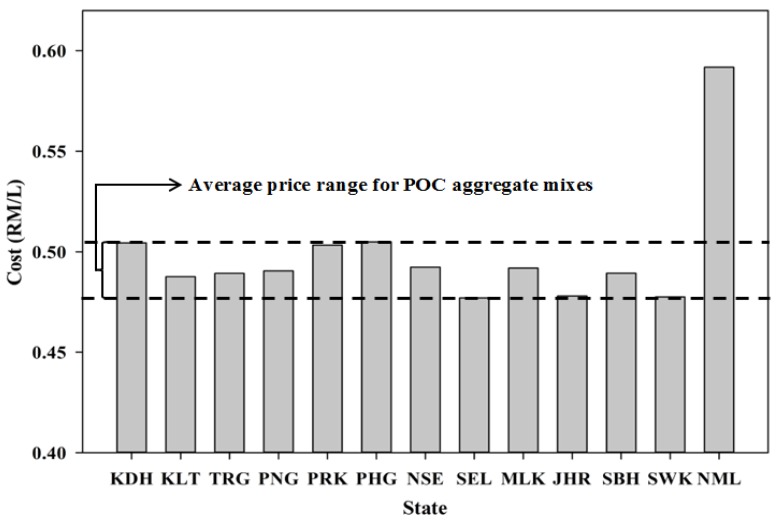
Cost of mortar incorporating POC aggregate (Kanadasan and Abdul Razak [32]).

In addition, the economic index of utilizing POC instead of other materials is very encouraging. Figure 18 shows the cost of the mortar specimens required for each state based on the mix design proportion. The cost or price data were obtained from a study carried out by Kanadasan and Abdul Razak [32]. A normal mix with river sand was used as a basis for comparison with all the POC mixes. As observed from the figure, the cost of the POC mortar specimens is much lower than the ordinary mixes. Almost 17% of the cost can be saved through the production of POC mortar specimens. Incorporating POC into the construction industry enhances the cost saving factor as well as introduces green building components to elevate the engineering economy index. Potential users that opt for a reduction in cost with a significant environmental component input may greatly benefit through its incorporation.

**Figure 19:**
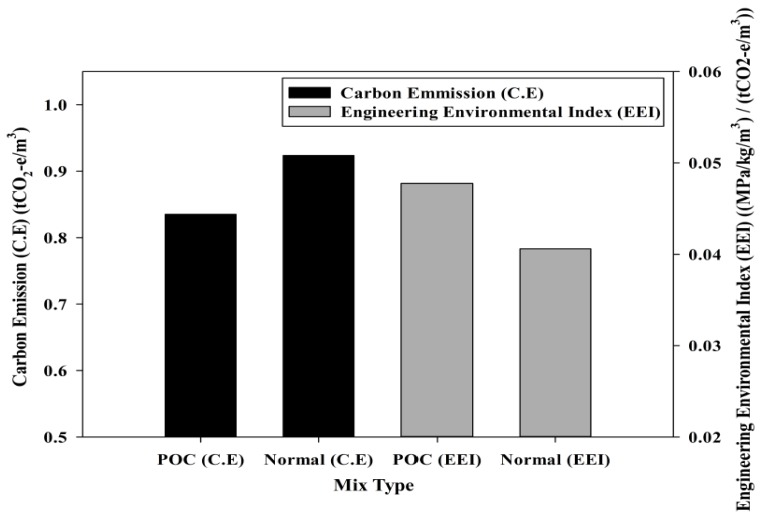
Relationship between carbon emission, mix type and engineering environmental index (Mineral Products Association [47], Department of Energy and Climate Change (DECC) [48]).

The carbon emission of mixtures incorporating POC will also have a positive outcome since there is an overall reduction. This will ensure a clean and safer environment. Studies have been carried out to determine the carbon emission for samples incorporating POC based on Mineral Products Association [47]. The analysis factors due to transport emissions was obtained from Department of Energy and Climate Change (DECC) [48]. Both of them were integrated into a series of analysis considering the materials that were used in this research and transportation requirements to produce a carbon emission data. Figure 19 shows the relationship between carbon emissions, mix type and Engineering Environmental Index (EEI). The incorporation of POC aggregate generally lowers the carbon footprint. On average, POC mixes from all the states in Malaysia produced a carbon emission of 0.84 tCO_2_-e/m^3^. However, the normal mix without the inclusion of POC had a carbon emission of 0.92 tCO_2_-e/m^3^ implying that the POC mixes lower the emission by 9.6%. Since the POC emission factor is very much lower compared to the normal mix, this proves that the utilization of POC gives a positive outcome. Taking into consideration the structural efficiency, the lower carbon emissions of POC also enhances the overall potential for it to be utilized in the construction industry. In this study, the EEI value due to POC aggregate incorporation was 17.6% higher than the normal mortar and thus enhances and promotes sustainability in the construction industry. This shows that POC can be utilized completely adopting a “zero-waste” concept to supplement the need for aggregate materials in mortar and concrete.

## 4. Conclusions

From this study, some vital consideration and information can be gathered to evaluate the feasibility of utilizing POC in the construction industry. The outcome of this study would provide a guideline on the characteristics of POC, engineering performance and their sustainability properties.

(1) Based on the overall characterization test carried out on each sample collected throughout each state in Malaysia, it can be concluded that the variation is not great and most of them fall in a narrow range.
(2) The satisfactory results obtained through the fresh and hardened properties test indicate the suitability of POC to be used as aggregate and in later stages as a binder material. The structural efficiency is within a range between 0.035 and 0.05 MPa/(kg·m^−3^), which is similar to normal mortar. The flexural strength of POC in this study is between 10 and 15 MPa. This process of utilizing waste materials from the agricultural industry also contributes to a better waste management system in addition to enhance the sustainability of the construction industry.
(3) The engineering and environmental features were also on the positive side with respect to the amount of greenhouse gas emissions, energy consumption and economic performance. POC can reduce the cost of construction by 17% compared to ordinary aggregates. The carbon emission and EEI value of POC was lowered by 9.6% and 17.6%, respectively. This indicates the potential of POC to replace the normal sand at a higher substitution rate.

Both the agricultural and construction industries could benefit from this waste utilization, as it allows for an improved waste management system by converting them into a useful product for building applications. Despite there being small variations for each state due to several factors, all the samples produced satisfactory results for the physical and chemical properties and are suitable for incorporation in both mortar and concrete. In future, this study could serve as a guide for upcoming research by providing sufficient information concerning the characteristics and suitability of POC to be utilized in the construction industry.
